# EFFECT OF INSPIRATORY MUSCLE TRAINING ON INSPIRATORY MUSCLE STRENGTH IN ADULTS WITH POST-COVID-19 CONDITION AND INSPIRATORY MUSCLE WEAKNESS: A RANDOMIZED CONTROLLED TRIAL

**DOI:** 10.2340/jrm.v58.44931

**Published:** 2026-04-21

**Authors:** Anna TÖRNBERG, Anna SVENSSON-RASKH, Elisabeth RYDWIK, Alexandra HALVARSSON, Judith BRUCHFELD, Malin NYGREN-BONNIER

**Affiliations:** 1 Division of Physiotherapy, Department of Neurobiology, Care Sciences and Society, Karolinska Institutet, Huddinge; 2 Medical Unit Allied Health Professionals, Women’s Health and Allied Health Professionals Theme, Karolinska University Hospital, Stockholm; 3 Division of Infectious Diseases, Department of Medicine Solna, Karolinska Institutet, Stockholm; 4 Department of Infectious Diseases, Theme Emergency and Reparative Medicine, Karolinska University Hospital, Stockholm, Sweden

**Keywords:** maximal inspiratory pressure, physical exercise, post-COVID-19 condition, respiratory muscles, respiratory muscle training

## Abstract

**Objective:**

Evaluate the effect of inspiratory muscle training in adults with post-COVID-19 condition (PCC) and inspiratory muscle weakness.

**Design:**

Randomized controlled trial.

**Subjects/Patients:**

Adults with PCC and inspiratory muscle weakness.

**Methods:**

Participants were randomized to inspiratory muscle training twice daily plus individualized exercise twice weekly or exercise alone, with weekly follow-ups over 8 weeks. Primary outcome: inspiratory muscle strength (Maximal Inspiratory Pressure). Secondary outcomes: expiratory muscle strength (Maximal Expiratory Pressure); functional capacity (Six‑Minute Walk Test; One‑Minute Sit‑to‑Stand); lung function (spirometry); dyspnoea (mMRC); respiratory symptoms (chest tightness, impaired deep breathing, breathing‑ related pain); cough frequency (CAAT cough item); fatigue (Fatigue Severity Scale); physical activity (Frändin–Grimby Activity Scale); activity limitations (Patient‑Specific Functional Scale); and health- related quality of life (EQ‑5D‑5L). Intention-to-treat analyses used imputed missing data. Estimated sample size: 90.

**Results:**

Forty-four participants were included (median age 47; 82% women; *n* = 22/group). Between‑group differences favoured the intervention for inspiratory muscle strength (mean difference: 18%; 95% CI: 5–30; OR for clinically meaningful improvement: 7.08, 95% CI: 1.31–38.32) and cough frequency. No other between-group differences were observed.

**Conclusion:**

Inspiratory muscle training may improve inspiratory muscle strength and reduce cough frequency, but limited sample size and underrepresentation of the most severely affected warrant cautious interpretation.

## LAY ABSTRACT

Some people with post-COVID-19 condition experience breathing difficulties and weakness in the inspiratory muscles (muscles used to inhale). This study tested whether training these muscles could help improve muscle strength and symptoms. Participants were randomly divided into 2 groups:1 performed inspiratory muscle training twice daily and personalised physical exercise twice weekly, while the other performed the physical exercise programme alone. Both groups met with a physiotherapist once a week for 8 weeks. The group that trained breathing muscles improved more in inspiratory muscle strength and reported less frequent coughing. Other outcomes were similar between the groups. Because fewer people were enrolled than planned, and those with the most severe fatigue often could not participate, the results should be interpreted with some caution. These findings suggest that inspiratory muscle training may help people with post-COVID-19 condition and weak breathing muscles to strengthen their inspiratory muscle strength and reduce cough frequency.

Post-COVID-19 condition (PCC), as defined by the World Health Organization (WHO), refers to symptoms that persist or develop after COVID-19, usually 3 months from onset and lasting at least 2 months without alternative diagnosis (1). PCC affects 1–7% of individuals who have experienced varying degrees of COVID-19 severity, and it may impact hundreds of millions globally and hundreds of thousands in the Nordic countries (2–5). PCC is a complex multisystem condition in which respiratory involvement is common and often coexists with sequelae from other organ systems, such as fatigue and post-exertional symptom exacerbation (PESE) (2). Respiratory manifestations may include reduced diffusion capacity of the lung for carbon monoxide, interstitial lung abnormalities, impaired inspiratory muscle strength, abnormal breath-ing pattern, and dyspnoea (2, 3, 6–9). Risk factors for respiratory sequelae in PCC include pre-existing chronic respiratory diseases, female sex, smoking, and severe acute COVID-19 illness (8).

Inspiratory muscle weakness in PCC may result from severe initial pneumonia requiring intensive care and mechanical ventilation (3, 9). In individuals with symptomatic PCC who did not have severe lung disease during COVID-19, inspiratory muscle weakness has been reported in nearly half within 1 year after COVID-19 (6). Potential causes include viral persistence in the lungs and respiratory muscles, autoimmunity, deconditioning, and autonomic dysfunction, which may coexist with underlying structural organ impairment (3, 8, 9). These factors can impair diaphragm activation and respiratory muscle strength, disrupt the breathing pattern, increase the effort of breathing, and trigger the respiratory muscle metaboreflex (6, 7, 9–11). Consequently, individuals may experience respiratory symptoms such as dyspnoea, reduced ability to take deep breaths, chest tightness, and cough, as well as extrapulmonary symptoms including palpitations, peripheral muscle weakness, and exercise intolerance (6, 9, 11).

Rehabilitative interventions have demonstrated promising results in PCC. Among these, inspiratory muscle training (IMT) implemented as part of rehabilitation, has emerged as a feasible and effective approach for managing respiratory symptoms in this population (2, 12). Reported benefits include improvements in inspiratory muscle strength, dyspnoea, diaphragm function, physical performance, exercise capacity, health-related quality of life (HRQoL), and physical activity (11, 13–16). These effects may not result solely from increased respiratory muscle strength per se, but also from improved autonomic regulation, breathing pattern, diaphragmatic activation, and modulation of the respiratory metaboreflex (10, 11). While IMT has shown promising results in PCC, its specific effects in those with inspiratory muscle weakness remain unexplored. Expanding our understanding of targeted interventions may support more effective rehabilitation strategies in PCC.

This study aimed to evaluate the effect of IMT, in addition to physical exercise, on inspiratory muscle strength in individuals with PCC and inspiratory muscle weakness. We hypothesised that IMT would be an effective intervention, leading to improvements in inspiratory muscle strength.

## METHODS

### Study design and setting

This assessor-blinded, two-armed randomized controlled trial (RCT) was conducted within the ReCOV research project (Recovery and rehabilitation during and after COVID-19), an observational cohort of hospitalized and non‑hospitalizsed individuals with PCC (17, 18). This RCT was initiated after the main project had started, based on observations of impaired inspiratory muscle strength, had its own protocol, was approved under the ReCOV ethical permission, and was prospectively registered at ClinicalTrials.gov (NCT05024474). Participants in ReCOV are primarily recruited from the specialized, interprofessional post-COVID outpatient clinic at Karolinska University Hospital, Stockholm, Sweden. At this clinic, assessments are conducted for adults who were referred following hospitalization due to COVID-19, as well as for non-hospitalized individuals referred from primary care or self-referred due to persistent symptoms (≥ 3 months) consistent with the WHO’s definition of PCC (1). For non-hospitalized individuals, referral also required a local clinical criterion of at least 50% ongoing sick leave or a new functional impairment following confirmed or suspected COVID‑19.

### Participants

For the present study, participants with PCC were recruited from the post-COVID outpatient clinic and/or by physiotherapists at primary care units in Stockholm. All individuals attending the post-COVID clinic were invited to participate in the ReCOV project; in parallel, eligibility for this RCT was assessed based on inspiratory muscle strength. Screening for eligibility was performed using the maximal inspiratory pressure (MIP) testing at the post-COVID clinic. In primary care, initial screening was based on symptoms suggestive of impaired inspiratory muscle strength (e.g., abnormal breathing pattern, impaired deep breathing, dyspnoea, chest tightness, and/or frequent yawning). All individuals who passed symptom screening in primary care underwent MIP testing to confirm eligibility.

Inclusion criteria were a diagnosis of PCC, age ≥ 18 years, and impaired inspiratory muscle strength. Inspiratory muscle strength was considered impaired if the MIP value was ≤ 80% of the lower limit of the 95% confidence interval for the predicted value (19). Participants were excluded if they had physical or cognitive impairments preventing participation in measurements or interventions, were currently undergoing or had recently completed an IMT intervention (< 3 months) or did not speak English or Swedish. Data were collected between September 2021 and April 2024.

### Randomization and blinding

Participants were randomized in a 1:1 ratio to either the intervention or active control group using random permuted blocks with variable block sizes (2, 4, and 6) in random order, based on a computer-generated randomization list. Group allocation was concealed in opaque envelopes, which were opened only after completion of the baseline assessments, making baseline assessors blinded to group allocation during testing. Outcome assessors conducting post-intervention measurements were also blinded to group allocation. All assessors had received training and were experienced in performing the assessments.

### Intervention

**Fig. 1:**
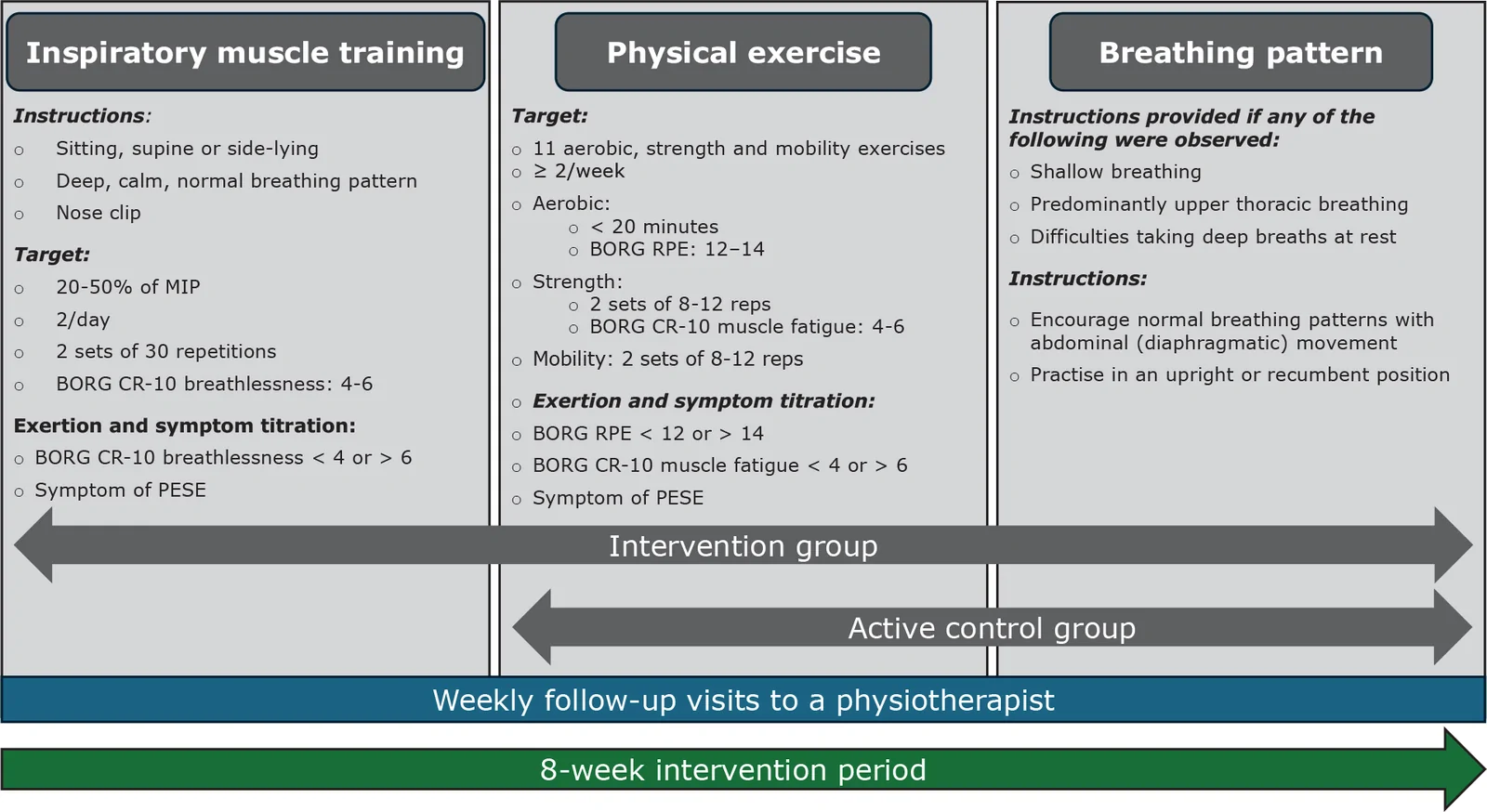
Description of the interventions. Overview of the intervention regimens for the intervention and active control group during the 8-week intervention period, including descriptions of progression and regression through adjustments in training volume (number of exercises, repetitions, or sets) and/or intensity. These adjustments were based on the presence of post-exertional symptom exacerbation (PESE) or exertion level.

The intervention regimens for the intervention and active control groups are detailed in Fig. 1. In summary, the intervention group was instructed to perform IMT twice daily, at home, using 1 of 2 pressure-regulated devices, selected based on each participant’s absolute baseline MIP value. All but 1 participant used the Threshold IMT (Philips Respironics, Murrysville, PA, USA), while 1 participant with a higher baseline MIP value used the POWERbreathe K3 (POWERbreathe International Ltd, Southam, UK), which allows for greater resistance. Participants in both groups receiv-ed instructions on normal breathing pattern when indicated, with guidance to incorporate abdominal (diaphragmatic) movement.

Both groups were instructed to follow an individualized physical exercise programme at least twice per week and received 1 programme adapted to a gym environment and 1 for home (see Appendix S1). Participants selected their preferred exercise setting (home, gym, or physiotherapy outpatient clinic) and could alternate settings across sessions. The exercise programmes were aligned in structure and training goals across settings – comprising aerobic warm‑up, multi‑joint strength exercises targeting major muscle groups and mobility/stretching – while specific exercises were adapted to available equipment (e.g., machines/free weights in the gym vs resistance bands/bodyweight at home). Intensity targets and progression were standardized across settings (see Fig. 1 and Appendix S1).

Resistance, intensity, and training volume for IMT and strength training were individually adjusted throughout the intervention based on the presence of PESE and exertion levels, guided by Borg RPE and CR-10 scales, in line with predefined criteria (Fig. 1 and Appendix S1) (20). The intervention period lasted 8 weeks and included at least 1 follow-up visit per week to a physiotherapist at either Karolinska University Hospital or a primary care unit, where the intervention was delivered as part of routine clinical care. The initial visit was always conducted on-site, during which participants were instructed and familiarized with the individually tailored IMT and exercise protocols. At subsequent weekly follow-up visits, adjustments were made as needed to the participants’ technique, exercise intensity, and/or volume based on the criteria outlined in Fig. 1. These visits were conducted either on-site or remotely (via telephone or video), depending on participant preference. Adherence and progression were monitored using training diaries and during follow‑up visits. The exact location of each exercise session was not recorded, and adherence was therefore summarized at the participant level.

### Outcomes

All outcomes were assessed by experienced physiotherapists at baseline and post-intervention at the post-COVID outpatient clinic. Additionally, demographic data including anthropometric measurements, socioeconomic status, medical history, clinical characteristics, and medications were collected through interviews and medical records. While demographic data were retrieved from the main ReCOV database, all intervention-related outcomes were specific to this study.

*Primary outcome.* The primary outcome was the change in inspiratory muscle strength, assessed using the MIP test and expressed as percentage of predictive value (19). Additionally, to evaluate clinical significance, we applied the recently established minimal clinically important difference (MCID) for MIP (≥ 22.1%), which corresponds to self-reported improvement in overall health among individuals with PCC after a respiratory muscle training programme (21). The MIP test is a non-invasive and clinically applicable method of global inspiratory muscle strength, which measures the maximum static inspiratory pressure at the mouth, that an individual can generate during inhalation (22). The test can be used to identify individuals with inspiratory muscle weakness and is responsive to evaluate within-subject change if performed according to guidelines. The participants were instructed to inhale forcefully from residual volume in a sitting position, using a nose clip and a hand-held Respiratory Pressure Meter device (MicroRPM) with a flanged mouthpiece, and holding a sustained pressure for at least 1.5 s (22). The physiotherapists leading the assessments gave clear instructions and encouragement to optimize the participant’s effort, including coaching to prevent air leaks around the mouthpiece. The best value from at least 3 attempts, which differed by less than 10%, was used as recommended by current guidelines. Participants received initial familiarisation with the device and procedure during their first clinical screening visit and were further allowed to familiarize themselves with the equipment during the baseline test session.

*Secondary outcomes.* All secondary outcomes were predefined to capture broader intervention effects and are listed below with corresponding assessment methods.

- Expiratory muscle strength: maximal expiratory pressure (MEP), percentage of predicted value (22).
- Functional capacity: Six-Minute Walk Test (6MWT) and One-Minute Sit-to-Stand Test (1-min STS), percentage of predicted values (23–25).
- Lung function: dynamic spirometry, percentage of predicted values for forced expiratory volume in 1 s (FEV_1_), forced vital capacity (FVC), and peak expiratory flow (PEF) (26).
- Dyspnoea: modified Medical Research Council scale (mMRC), (0–4; higher scores indicate more severe dyspnoea) (27).
- Self-reported respiratory symptoms: chest tightness, difficulty taking deep breaths, and breathing‑related pain (binary: present/absent).
- Cough frequency: Chronic Airways Assessment Test (CAAT) cough item (1–5; higher scores indicate more frequent cough) (28).
- Fatigue: Fatigue Severity Scale (FSS), (FSS mean score: 1–7; higher scores indicate more severe fatigue) (29).
- Physical activity: Frändin–Grimby Activity Scale (1–6; higher scores indicate higher activity level) (30).
- Activity limitations: Patient‑Specific Functional Scale (PSFS), (PSFS mean score: 0–10; higher scores indicate less limitation in performing 3 self-selected activities) (31).
- HRQoL and self-rated health: EQ‑5D‑5L (EQ‑5D index 0.243 to 1.000; EQ VAS 0–100) (32). The MCID for EQ VAS in PCC was predefined as ≥ 7.5 (33).

### Sample size calculation

Sample size was calculated for a two‑sided *t*‑test (α = 0.05, power = 80%), assuming a 10‑percentage‑point between‑group difference in MIP (% predicted) and a moderate effect size (Cohen’s *d* = 0.6) (34). This corresponded to a required sample size of 36 participants per group. Allowing for 25% dropout, 45 participants per group were planned (total *n* = 90).

## DATA MANAGEMENT AND ANALYSIS

Data were managed using REDCap electronic data capture tools hosted at Karolinska Institutet and all statistical analyses were performed using RStudio (R Foundation for Statistical Computing, Vienna, Austria) (35, 36). Descriptive statistics were reported as median and interquartile range (IQR) for continuous and ordinal variables, due to the relatively small sample size in each group, and as number of cases out of valid observations (*n*/*N*) with corresponding percentage (%) for binary variables, based on observed data. Normality and variance assumptions were assessed using Shapiro- Wilk and Levene’s tests.

Between-group differences in continuous and ordinal variables at baseline were analysed using independent samples *t*-tests when both normality and equal variance assumptions were met, Welch’s *t*-test when normality was met but variances were unequal, or Mann–Whitney *U* tests otherwise. Binary variables were analysed using χ^2^ or Fisher’s exact tests. Within‑group changes in continuous and ordinal variables between baseline and post-intervention were analysed using paired *t*‑tests when normality assumptions were met, or Wilcoxon signed‑rank tests otherwise. Binary symptom variables were analysed using McNemar’s test. MCID indicators (MCID for MIP and EQ VAS) were not subject to within‑group testing as they represent individual level change.

Between-group differences in outcome variables at post-intervention were assessed using ANCOVA for continuous and ordinal outcomes with more than 2 levels (treated as continuous), and logistic regression for binary outcomes. In all models, the post‑intervention value of the outcome was used as the dependent variable, and group allocation was included as the main independent variable, with baseline value of the outcome, sex, and age included as covariates. Model assumptions were checked both numerically and visually. The level of significance was set at *p* < 0.05 for all analyses. Secondary outcomes were considered exploratory; therefore, no adjustment for multiple testing was applied.

Missing post-intervention data were imputed using multiple imputation via the mice package in R (38), with baseline values, group allocation, sex, and age as predictors, and imputation methods tailored to variable type. The plausibility of the Missing At Random (MAR) assumption was assessed via logistic models predicting missingness from baseline and group. No outcome was associated with group allocation; only 6MWT and PSFS were associated with baseline values, supporting the MAR assumption.

All primary analyses were conducted using an intention-to-treat approach, with results pooled across 20 imputed datasets. To assess the robustness of the findings, a sensitivity analysis was performed using a complete case approach, including only participants with available post-intervention data. Between-group differences at baseline and within-group analyses were based on observed, non-imputed data.

## RESULTS

### Participants

**Fig. 2:**
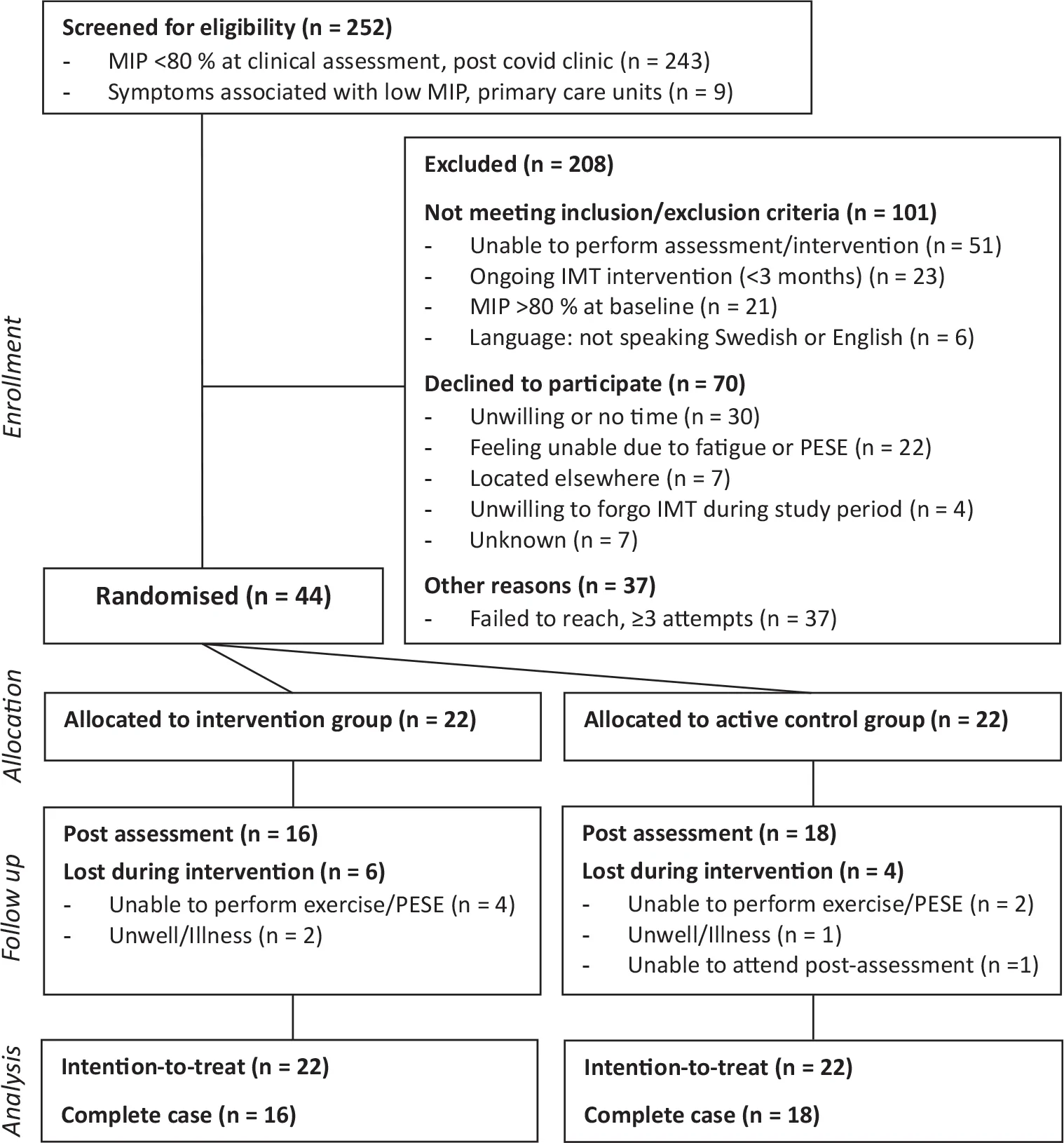
Participant flowchart.

Of the 252 individuals screened, 101 were excluded based on eligibility criteria, as shown in the flowchart in Fig. 2. Among the 151 eligible individuals, 44 could be enrolled, yielding a recruitment rate of 29%. Notably, 55 individuals who fulfilled the inclusion criterion of impaired MIP were excluded because they were unable to manage the assessment or the intervention, and 22 declined participation due to severe fatigue or PESE. Other exclusions or declinations were due to reasons not directly related to PCC. The 44 participants were randomized equally to the intervention and active control group (*n* = 22 in each). There were no significant differences in baseline characteristics between the intervention and active control groups (Table I), nor in outcome variables at baseline (all *p*-values > 0.05). The intervention period commenced within 1 month after the baseline assessments. During the study, 10 participants (23%) withdrew, primarily due to symptom exacerbation consistent with PESE following physical exercise (see Fig. 2). Withdrawn participants did not differ significantly from completers in most baseline characteristics, although they had significantly lower employment/study rate ≥ 50%, 6MWT, 1-min STS, PSFS, and EQ VAS (*p* < 0.05), as shown in Table SI.

**Table I:** Characteristics of participants in the intervention and active control group at baseline

Variable	Intervention group (*n* = 22)	Active control group (*n* = 22)	*p*- value
Demographics			
Age (years)	47 (39–58)	50 (44–56)	0.76
Sex (female)	18/22 (82%)	18/22 (82%)	1.00
BMI (kg/m^2^)	28 (24–29)	27 (23–29)	0.50
Higher education (> 12 years)	17/20 (85%)	14/19 (74%)	0.45
Previous work/study (≥ 50 %)	18/22 (82%)	17/22 (77%)	1.00
Current work/study (≥ 50 %)	13/22 (59%)	10/22 (46%)	0.55
Medical history			
Former/never smoker	21/22 (96%)	22/22 (100%)	1.00
No. previous comorbidities	2 (0–4)	2 (1–5)	0.95
Previous asthma	4/22 (18%)	2/22 (9%)	0.66
Previous COPD	0/22 (0%)	0/22 (0%)	–
Previous interstitial lung disease	0/22 (0%)	0/22 (0%)	–
Hospitalized due to COVID-19	6/22 (27%)	5/22 (23%)	1.00
Intensive care unit	4/22 (18%)	1/22 (5%)	0.35
First wave of transmission	8/22 (36%)	11/22 (50%)	0.54
Months since COVID-19	19 (16–24)	22 (15–28)	0.59
Clinical characteristics at baseline			
POTS/IST	2/22 (9%)	4/22 (18%)	0.70
No. of PCC symptoms	11 (8–13)	14 (9–15)	0.24
Self-reported symptom of PESE	17/22 (77%)	17/22 (77%)	1.00
FSS ≥ 4	17/19 (89%)	18/19 (95%)	1.00
mMRC dyspnoea ≥ 2	21/22 (96%)	21/22 (96%)	1.00
SpO2, at rest	100 (100–100)	100 (100–100)	0.20
Respiratory rate, at rest	14 (12–16)	12 (10–17)	0.36
Mucus	4/22 (18%)	9/22 (41%)	0.10
Heart rate, at rest	70 (66–78)	76 (68–84)	0.18
Inhalation, bronchodilator	6/22 (27%)	5/22 (23%)	1.00
Inhalation, anti-inflammatory	5/22 (23%)	5/22 (23%)	1.00
Beta blocker	6/22 (27%)	7/22 (32%)	1.00

Data are presented as median (IQR), or as number of cases out of valid observations (*n*/*N*) with corresponding percentages (%). Group differences were assessed using independent samples *t*-tests, Welch’s *t*-test, Mann–Whitney *U* tests, χ^2^ tests, or Fisher’s exact tests, as appropriate, with *p*-values reported.

COPD: chronic obstructive pulmonary disease; FSS: Fatigue Severity Scale; IST: inappropriate sinus tachycardia; mMRC: modified Medical Research Council dyspnoea scale; PCC: post-COVID-19 condition; PESE: post-exertional symptom exacerbation; POTS: postural orthostatic tachycardia syndrome.

Sixteen of 22 participants in the intervention group and 18 of 22 in the active control group completed the study. Among intervention group completers, 9 (56%) performed IMT twice daily, and 14 (88%) at least once daily. Physical exercise was performed twice weekly by 10 participants in each group (63% and 56%, respectively), and at least once weekly by 15 participants (94% and 83%). Four participants per group (25% and 22%) reduced exercise volume/intensity due to PESE, reporting increased fatigue and respiratory/musculoskeletal symptoms. No serious adverse events were reported. Breathing pattern instructions were provided to 13 participants (81%) in the intervention group and 11 (61%) in the active control group. The number of physiotherapist follow-up visits was similar between groups, with a median of 7 (IQR 6–8) visits in both.

### Effect on primary outcome

**Fig. 3:**
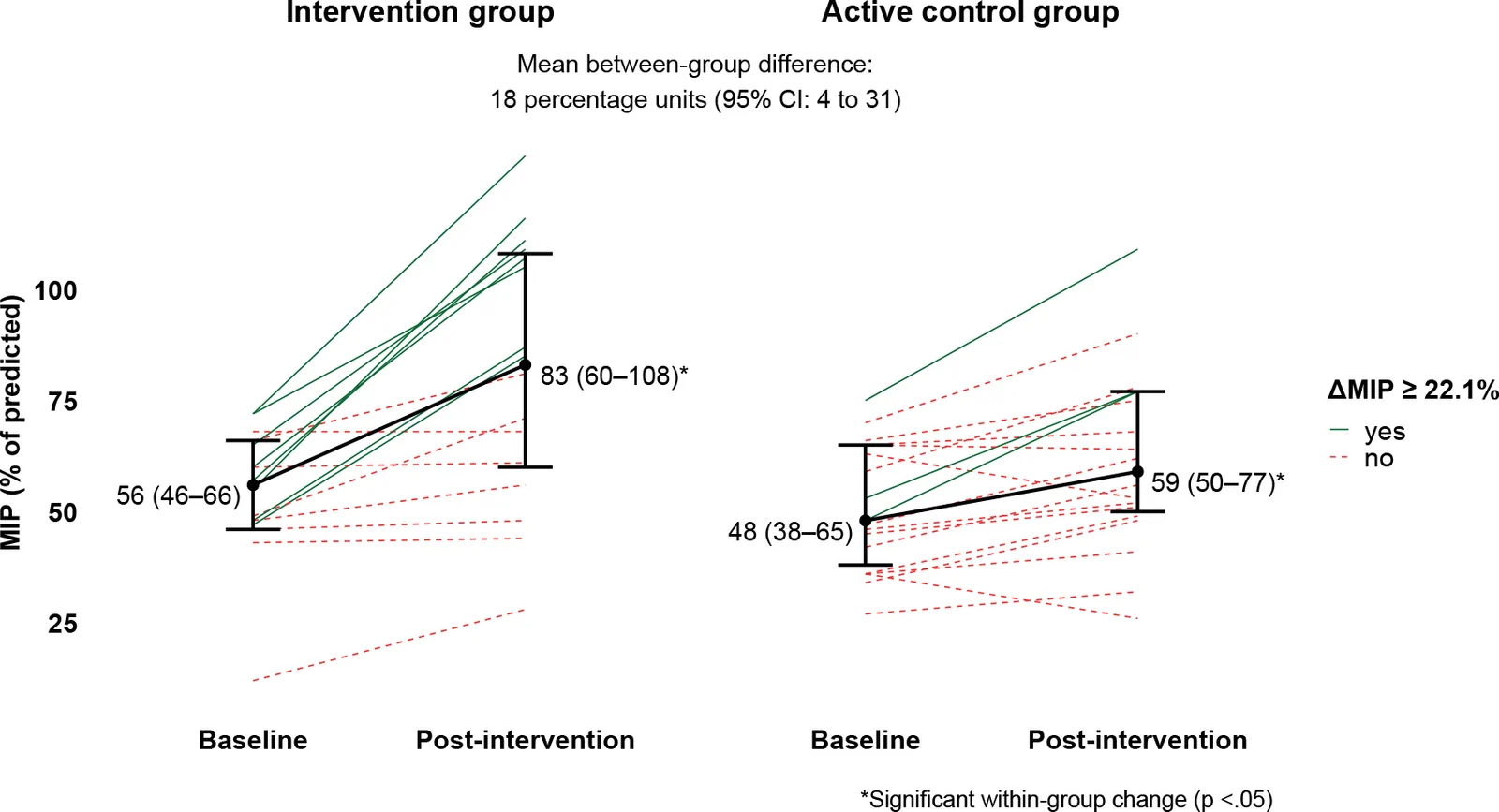
Between-group difference and within-group change in maximal inspiratory pressure (MIP) and clinically meaningful change. Between-group difference in MIP, based on imputed intention-to-treat analysis, and within-group changes in MIP from baseline to post-intervention in the intervention and active control group. Median (IQR) MIP values are shown for each group at both time points, along with mean within-group changes (black solid line; *n* = 22 in both groups). Statistically significant within-group changes are indicated with an asterisk (*). Individual trajectories among participants who completed the study (intervention group: *n* = 16, active control group: *n* = 18) are coloured and styled according to clinically meaningful improvement in MIP (ΔMIP ≥ 22.1%): green solid lines indicate participants who achieved a clinically important improvement, and red dashed lines indicate those who did not.

The increase in inspiratory muscle strength (MIP) was significantly greater in the intervention group compared with the active control group, with a mean between-group difference of 18 percentage points (95% CI: 5 to 30), as seen in Fig. 3. Within-group analyses showed statistically significant increases in inspiratory muscle strength (MIP) in both groups: an increase of 28 percentage points (95% CI: 16 to 40) in the intervention group, and 10 percentage points (95% CI: 3 to 17) in the active control group. Furthermore, responder analysis revealed that the odds of achieving a clinically meaningful improvement in inspiratory muscle strength (ΔMIP ≥ 22.1%) were significantly higher in the intervention group, with an odds ratio of 7.08 (95% CI: 1.31 to 38.32). In the intervention group, 8 of 16 participants (50%) exceeded the MCID, compared with 3 of 18 (17%) in the active control group.

### Effect on secondary outcomes

For cough frequency, assessed using the cough item of the CAAT, a statistically significant between-group difference was observed, favouring the intervention group (mean difference –0.6, 95% CI –1.1 to –0.1; Table II). No other statistically significant between-group differences were found across the remaining secondary outcomes.

**Table II:** Between-group difference and within-group change in secondary outcomes

Outcome	Group	Baseline	Post-intervention	Adj. between-group diff b (95% CI)
Continuous				
MEP (% pred)	Intervention	63 (51–86)	74 (59–93)	12 (–5; 29)
	Active control	67 (51–86)	68 (47–92)	
6MWD (% pred)	Intervention	83 (62–93)	94 (77–110)	7 (–4; 18)
	Active control	78 (67–93)	82 (64–96)	
1-min STS (% pred)	Intervention	49 (39–68)	71 (48–88)**	10 (–2; 22)
	Active control	45 (43–57)	56 (48–64)	
FEV1 (% pred)	Intervention	85 (72–95)	83 (77–96)	–2 (–10; 5)
	Active control	89 (78–93)	94 (82–99)	
FVC (% pred)	Intervention	85 (73–90)	84 (81–90)	–3 (–10; 5)
	Active control	81 (75–91)	86 (81–100)	
PEF (% pred)	Intervention	70 (63–87)	81 (51–86)	–3 (–13; 6)
	Active control	78 (70–82)	76 (69–85)	
Dyspnoea (mMRC score: 0–4)	Intervention	2 (2–2)	2 (1–2)	–0.3 (–0.8; 0.2)
	Control	2 (2–3)	2 (2–3)	
CAAT cough item (score: 1–5)	Intervention	2 (1–3)	1 (1–2)	–0.6 (–1.1; –0.1)*
	Control	2 (1–2)	2 (1–3)	
FSS (mean score)	Intervention	6.2 (5.2–6.8)	5.4 (4.2–6.4)	–0.4 (–1.2; 0.3)
	Active control	6.0 (5.5–6.7)	5.8 (4.8–6.2)	
Frändin–Grimby (score: 1–6)	Intervention	3 (2–3)	3 (3–4)	–0.1 (–0.5; 0.3)
	Active control	3 (2–3)	3 (2–4)	
PSFS (mean score)	Intervention	3.00 (2.00–3.33)	5.17 (4.00–6.42)**	1.03 (–0.48; 2.55)
	Active control	2.33 (1.33–3.00)	3.33 (1.67–5.67)	
EQ-5D index	Intervention	0.761 (0.632–0.854)	0.814 (0.766–0.896)	0.010 (–0.081; 0.101)
	Active control	0.727 (0.629–0.812)	0.789 (0.649–0.844)	
EQ VAS	Intervention	55 (36–70)	65 (60–75)**	7 (–3; 16)
	Active control	50 (26–65)	55 (38–70)	

Binary				OR (95%CI)

Chest tightness	Intervention	17/22 (77%)	9/16 (56%)	0.98 (0.20; 4.80)
	Active control	18/22 (82%)	11/18 (61%)	
Impaired deep breathing	Intervention	19/22 (86%)	12/16 (75%)	0.98 (0.16; 6.04)
	Active control	18/22 (82%)	14/18 (78%)	
Breathing-related pain^a^	Intervention	5/22 (23%)	4/16 (25%)	5.88 (0.52; 66.48)
	Active control	9/22 (41%)	3/18 (17%)	
MCID EQ VAS (ΔEQ VAS ≥ 7.5)	Intervention	–	7/16 (44%)	1.63 (0.30; 9.02)
	Active control	–	8/18 (44%)	

Secondary outcomes at baseline and post-intervention, including within- and between-group analyses. Data are presented as median (IQR), or as number of cases out of valid observations (*n*/*N*) with corresponding percentages (%). Within-group changes were assessed using paired *t*-tests, Wilcoxon signed-rank tests or McNemar’s tests, based on observed, non-imputed data. Between-group differences were analysed using ANCOVA or logistic regression. Group allocation was included as the main independent variable in all models, with baseline value of the outcome, sex, and age included as covariates unless otherwise stated. The results are reported as unstandardized regression coefficients (b) or odds ratios (OR) with 95% confidence intervals (CI).

a Sex excluded in model due to the near absence of breathing pain in male participants, causing model instability from quasi-complete separation.

* Statistically significant between-group difference (*p* < 0.05).

** Statistically significant within-group change (*p* < 0.05).

1-min STS: 1-minute sit-to-stand test; 6MWT: 6-minute walk test; CAAT: Chronic Airways Assessment Test; EQ-5D index: EuroQol 5-Dimension index; EQ VAS: EuroQol visual analogue scale; FEV1: forced expiratory volume in 1 s; F/G: Frändin–Grimby activity score; FSS: Fatigue Severity Scale; FVC: forced vital capacity; MCID: minimal clinically important difference; MEP: maximal expiratory pressure; mMRC: modified Medical Research Council dyspnoea scale; PEF: peak expiratory flow; PSFS: Patient-Specific Functional Scale; Δ: change.

Within-group analyses revealed statistically significant improvements in the intervention group for the 1-min STS (mean change = 15 percentage points; 95% CI: 5 to 25), PSFS mean score (mean change = 1.74; 95% CI: 0.58 to 2.91), and EQ VAS (mean change = 9; 95% CI: 1 to 17). The control group showed no significant within-group changes in any secondary outcomes.

### Sensitivity analyses

Sensitivity analysis using a complete case approach for both primary and secondary outcomes yielded results like those of the intention-to-treat analysis, both in direction and in magnitude of effect estimates.

## DISCUSSION

This RCT showed that adding IMT to physical exercise could lead to statistically and possibly clinically meaningful improvements in inspiratory muscle strength (MIP) among individuals with PCC and inspiratory muscle weakness who were able to participate. A significant between‑group difference was also observed for cough frequency (CAAT cough item). No other outcomes differed between groups. Given the substantially lower sample size than planned, all findings should be interpreted with caution and may primarily be applicable to individuals able to tolerate structured rehabilitation rather than those with more severe symptom burden.

Although the mean between-group difference in inspiratory muscle strength (MIP) fell short of the MCID, the 95% confidence interval encompassed it, suggesting the potential for a clinically meaningful effect. This interpretation is further supported by the responder analysis, which showed significantly higher odds of achieving a clinically meaningful effect in the intervention group. Moreover, within-group analyses indicated that only the intervention group achieved a clinically relevant increase in MIP (21). The increase in MIP observed in the active control group may be explained by effects of the physical exercise programme and natural recovery over time. Additionally, learning effects related to repeated MIP testing may have contributed. The relatively wide confidence intervals reflect a degree of uncertainty in the estimates, likely due to the modest sample size, substantial variability in MIP change, and potentially varying adherence to the interventions. Our findings are consistent with previous studies on IMT in PCC, conducted 9–22 months after COVID-19, demonstrating similar between-group differences in MIP (13, 15, 16). Unlike those studies, where participants had baseline MIP values within or slightly below the normal range (79–92% of predicted), the present study extends the current evidence base by demonstrating that IMT may also be beneficial in individuals with PCC and substantially reduced inspiratory muscle strength.

Between‑group differences in secondary outcomes were generally absent, with the exception of cough frequency (CAAT cough item), which favoured the intervention group. The intervention group demonstrated within‑group improvements in functional capacity (1‑min STS), activity limitations (PSFS), and self‑ rated health (EQ VAS), whereas no such changes were observed in the active control group. Improvements in the 1‑min STS observed in the intervention group are directionally consistent with previous studies (10, 13) and may align with the hypothesis that IMT reduces respiratory metaboreflex activity, thereby improving peripheral muscle perfusion and supporting functional task performance (10). The observed improvement in self‑rated health (EQ VAS) in the intervention group (ΔEQ VAS: 9; 95% CI: 1 to 17) exceeded the MCID (≥ 7.5) at the group level, but the wide confidence interval, including values below the threshold, limits the certainty of its clinical relevance (33). This potential effect on self‑rated health aligns with previous findings in individuals with PCC (13, 16). The absence of between‑group differences for other secondary outcomes may reflect limited statistical power rather than a definitive lack of effect. Nonetheless, given the absence of corresponding between‑group differences, the exploratory nature of these analyses, and the lack of correction for multiple testing, within‑group findings should be interpreted with caution.

Improvements in respiratory symptoms reported by participants in both groups suggest that IMT and/or physical exercise may positively influence symptom burden, consistent with earlier findings in individuals with PCC (11, 13–15). However, causal attribution is limited by the absence of an inactive control group, and spontaneous recovery over the 8‑week intervention period cannot be fully excluded, despite the prolonged symptom duration of the study population.

Adherence to the prescribed twice-daily IMT was moderate, although most participants completed at least 1 session per day. Adherence to physical exercise showed similar patterns across groups: moderate at the prescribed frequency, but higher when considering participation at least once per week. Despite challenges with full adherence, most participants engaged meaningfully, potentially contributing to the observed effects. The reported rates of full adherence to the interventions were generally lower than those observed in previous IMT studies in PCC (13–15). These discrepancies may reflect differences in training protocols, such as frequency and the use of supervised vs unsupervised sessions, as well as variations in study populations. Notably, our sample comprised individuals with a high symptom burden and prevalence of PESE, perhaps not included in previous studies.

These findings have several clinical implications for the design and delivery of rehabilitation interventions in individuals with PCC. Although IMT combined with physical exercise may offer benefits for some, the feasibility of this combined approach appears limited in this population, as reflected by the low recruitment rate and relatively high dropout rate observed. This suggests that the feasibility and potential effects may not extend to individuals with more substantial functional limitations, including those with severe fatigue or PESE. The heterogeneity and complexity of PCC underscore the challenges involved in developing effective interventions for this population. Although several individuals with PESE declined participation, 77% of enrolled participants reported PESE, indicating that those with milder PESE could still engage with individualized IMT-based interventions. Careful diagnostic procedures, comprehensive assessment, individual tailoring, and ongoing adaptations in clinical care are essential to ensure appropriate targeting, as supported by previous research (39, 40). IMT as monotherapy may be more feasible and relevant for individuals with PESE who have limited tolerance for physical exertion (39, 40). However, this hypothesis requires further investigation. Future research should focus on individually tailored interventions that account for factors such as PESE when addressing respiratory manifestations in PCC and consider participants’ perspectives to better understand acceptability alongside clinical effects.

Several methodological limitations warrant consideration. Recruitment ceased once it became clear that reaching the intended sample size within a reasonable timeframe was not feasible. Recruitment patterns indicate a risk of selection bias. Participants with the most severe fatigue, PESE, or functional limitations were less likely to enrol or remain, which limits representativeness and generalizability. The substantially smaller‑than‑planned sample size reduced statistical power and may have contributed to wide confidence intervals, warranting cautious interpretation of effect estimates. Withdrawals, who had lower baseline values in some variables, may have biased results by overestimating effects among completers. Although complete-case sensitivity analyses showed similar patterns, residual risk remains. The limited sample size necessitated an adaptation of the original analysis plan to maintain statistical robustness. As a result, ANCOVA was used instead of linear mixed models, and robust imputation techniques were employed to minimize bias (37, 38). Treating ordinal outcomes with more than 2 levels as continuous in the ANCOVA models reinforces the need for cautious interpretation. This approach was chosen because the sample size was insufficient for stable estimation using ordinal regression, and dichotomization would have resulted in loss of information. Exploratory analyses on secondary outcomes were not corrected for multiple testing, increasing type I error risk. Given the limited sample size, adherence was not included as a covariate, which may have influenced effect estimates and limits interpretation of dose–response relationships. Additionally, exercise sessions were conducted in varying settings, but session location was not recorded or adjusted for, which may have introduced unmeasured heterogeneity in training exposure and limiting the ability to fully isolate IMT‑specific effects.

Methodological rigour was ensured through randomized group allocation and blinded assessors. Blinding of participants and therapists was not feasible due to the nature of the intervention, a common challenge in rehabilitation research. Although sham-IMT could theoretically be used, true blinding is uncertain as resistance differences may be perceived. To assess inspiratory muscle strength in a clinically applicable manner, MIP was chosen. However, it is a measure reliant on voluntary effort and limited in clinical interpretability due to the wide range of normative values (19, 22). To address these limitations, we applied standardized testing procedures conducted by experienced assessors, used synthesized reference values derived from a diverse meta-analysis, and incorporated the recently established MCID for MIP in individuals with PCC (19, 21). To enhance clinical applicability, the study was designed to reflect routine practice by enabling remote follow-ups, supporting individually adapted interventions, and mainly using the only pressure-regulated IMT device available for clinical prescription at the time. Participants were recruited through standard care pathways, and physiotherapists delivered the intervention as part of their regular clinical work, with minimal disruption. Adherence and follow-up were, however, more closely monitored than in usual care to assess adherence and fidelity.

The sample was predominantly female, consistent with PCC epidemiology (8). Most participants were recruited from a specialized outpatient clinic and may represent a more severely affected subgroup, which may limit generalizability to the broader PCC population. At the same time, the most severely affected individuals may have been underrepresented despite the design intended to accommodate substantial fatigue and PESE, leaving generalizability to hard‑to‑reach individuals uncertain. The intervention was delivered 15–28 months post‑COVID‑19, reflecting referral criteria and clinical waiting times. It is unclear whether timing influenced the results in either direction. Finally, the relatively short intervention period and absence of long-term follow-up limit conclusions regarding sustained effects.

In conclusion, this study suggests that IMT, when added to physical exercise, may improve inspiratory muscle strength and reduce cough frequency in adults with PCC and inspiratory muscle weakness who were able to participate. No effects were observed for other outcomes, indicating a limited impact beyond respiratory-specific measures. Given the smaller than planned sample size, reduced statistical power, and likely underrepresentation of individuals with greater symptom burden, the findings should be interpreted with caution and considered exploratory. IMT may serve as a targeted component of tailored rehabilitation for selected subgroups, although further research is needed to establish its acceptability and broader clinical relevance.

## Supplementary Material




