# Supplementary material for: EFFECT OF INSPIRATORY MUSCLE TRAINING ON INSPIRATORY MUSCLE STRENGTH IN ADULTS WITH POST-COVID-19 CONDITION AND INSPIRATORY MUSCLE WEAKNESS: A RANDOMIZED CONTROLLED TRIAL
Source: J Rehabil Med. 2026 Apr 21;58:44931. doi: 10.2340/jrm.v58.44931 (PMC13107392; doi:10.2340/jrm.v58.44931)
Supplement: Supplementary file 1 [file JRM-58-44931-s1.pdf]

Supplementary material has been published as submitted. It has not been copyedited, or typeset by Journal of Rehabilitation Medicine

Individualised physical exercise program

General Guidelines

- Frequency: ≥ 2/week.
- **Aerobic exercise**:
- Intensity: Borg RPE 12–14 (reduce if experiencing PESE).
- Duration: ≤ 20 minutes (reduce if PESE).
- **Strength Training**:
- Intensity: Borg CR-10 4–6 (peripheral muscle fatigue). Reduce number of exercises, intensity, and/or reps/sets if PESE.
- 2 sets of 8–12 reps, 1-10 individually tailored exercises (e.g. equipment, body position).

Gym exercises:

1. Aerobic exercise and warm-up: Any aerobic exercise (e.g., cycling, walking)
2. Bicep Curl and Shoulder Press
3. Seated Leg Press
4. Seated Row
5. Lying Chest Press
6. Leg Extension
7. Seated Leg Curl
8. Lat Pulldown
9. Triceps Press
10. Mobility: Back rotation
11. Mobility: Back side stretch

Home exercises:

1. Aerobic exercise and warm-up: Any aerobic exercise (e.g., cycling, walking)
2. Seated or standing row
3. Squat
4. Bicep Curl
5. Lunge
6. Wall push-up
7. Calf-rise
8. Triceps press
9. Glute Bridge
10. Mobility: Back rotation
11. Mobility: Back side stretch
