# Supplementary material for: EFFECT OF INSPIRATORY MUSCLE TRAINING ON INSPIRATORY MUSCLE STRENGTH IN ADULTS WITH POST-COVID-19 CONDITION AND INSPIRATORY MUSCLE WEAKNESS: A RANDOMIZED CONTROLLED TRIAL
Source: J Rehabil Med. 2026 Apr 21;58:44931. doi: 10.2340/jrm.v58.44931 (PMC13107392; doi:10.2340/jrm.v58.44931)
Supplement: Supplementary file 2 [file JRM-58-44931-s2.pdf]

Supplementary material has been published as submitted. It has not been copyedited, or typeset by Journal of Rehabilitation Medicine

Table SI. Characteristics of participants who completed the study and those who withdrew.

Variable	Completers (n = 34)	Withdrawals (n = 10)	Difference
Demographics			
Age (years)	50 (39-59)	47 (40-53)	0.40
Sex (female)	28/34 (82%)	8/10 (80%)	1.00
BMI (kg/m^2)	28 (24-29)	25 (22-28)	0.31
Higher education (> 12 years)	23/31 (74%)	8/8 (100%)	0.17
Previous work/study (≥ 50 %)	29/34 (85%)	6/10 (60%)	0.17
Current work/study (≥ 50 %)	21/34 (62%)	2/10 (20%)	0.03*
Former/never smoker	34/34 (100%)	9/10 (90%)	0.23
No. previous co-morbidities	2 (0-4)	2 (2-8)	0.27
Previous Asthma	4/34 (12%)	2/10 (20%)	0.61
Previous COPD	0/34 (0%)	0/10 (0%)	–
Previous Interstitial Lung Disease	0/34 (0%)	0/10 (0%)	–
Hospitalised due to COVID-19	9/34 (26%)	2/10 (20%)	1.00
Intensive care unit	4/34 (12%)	1/10 (10%)	1.00
First wave of transmission	17/34 (50%)	2/10 (20%)	0.15
Months since COVID-19	21 (16-25)	21 (16-25)	0.92
Clinical characteristics and outcomes at baseline			
POTS/IST	4/34 (12%)	2/10 (20%)	0.42
No. of PCC symptoms	11 (9-14)	14 (12-18)	0.06
Self-reported symptom of PESE	26/34 (77%)	8/10 (80%)	1.00
mMRC dyspnoea ≥ 2	32/34 (94%)	10/10 (100%)	1.00
SpO2, at rest	100 (100-100)	100 (100-100)	0.13
Respiratory rate, at rest	14 (12-16)	13 (12-16)	0.90
Cough, (1=never, 5=all the time)	2 (1-2)	2 (1-3)	0.71
Mucus	11/34 (32%)	2/10 (20%)	0.70
Chest tightness	28/34 (82%)	7/10 (70%)	0.40
Impaired deep breathing	28/34 (82%)	9/10 (90%)	1.00
Breathing related pain	11/34 (32%)	3/10 (30%)	1.00
Heart rate, at rest	72 (67-81)	72 (65-79)	0.81
Inhalation, bronchodilator	9/34 (27%)	2/10 (20%)	1.00
Inhalation, anti-inflammatory	9/34 (27%)	1/10 (10%)	0.41
Beta blocker	10/34 (29%)	3/10 (30%)	1.00
MIP (% pred)	51 (45-65)	50 (36-65)	0.61
MEP (% pred)	70 (50-89)	55 (54-67)	0.22
6MWD (% pred)	84 (71-98)	69 (58-76)	0.02*
1-min STS (% pred)	49 (43-67)	43 (38-46)	0.049*
FEV1 (% pred)	87 (78-95)	87 (75-93)	0.92
FVC (% pred)	85 (77-90)	80 (71-94)	0.76
PEF (% pred)	75 (67-84)	74 (65-83)	0.96
FSS (mean score)	5.8 (5.3-6.5)	6.8 (6.5-7.0)	0.10
Frändin/Grimby activity score (1-6)	3 (3-3)	3 (2-3)	0.23
PSFS (mean score)	3 (2-4)	1.67 (1-2)	0.01*
EQ5D-index	0.771 (0.664-0.842)	0.641 (0.609-0.700)	0.17
EQ VAS	55 (40-70)	25 (23-34)	0.01*

Data are presented as median (IQR), or as number of cases out of valid observations (n/N) with corresponding percentages (%). Group differences were assessed using independent samples t-tests, Welch's t-test, Mann–Whitney U tests, Chi-square tests, or Fisher's exact tests, as appropriate, with p values reported.
*Statistically significant difference.
1-min STS: 1-minute sit-to-stand test; 6MWT: 6-minute walk test; COPD: Chronic Obstructive Pulmonary Disease; EQ5D-index: EuroQol 5-Dimension index; EQ VAS: EuroQol visual analogue scale; FEV_1: forced expiratory volume in 1 second; FSS: Fatigue Severity Scale; FVC: forced vital capacity; IST: Inappropriate Sinus Tachycardia; MEP: maximal expiratory pressure; mMRC: modified Medical Research Council dyspnoea scale; PCC: post-COVID-19 condition; PESE: Post-Exertional Symptom Exacerbation; PEF: peak expiratory flow; POTS: Postural Orthostatic Tachycardia Syndrome. PSFS: Patient-Specific Functional Scale
